# Reliability and validity study of the Thai adaptation of the Maslach Burnout Inventory-Student Survey among preclinical medical students at a medical school in Thailand

**DOI:** 10.3389/fpsyg.2023.1054017

**Published:** 2023-05-03

**Authors:** Wasit Wongtrakul, Yodying Dangprapai, Nattha Saisavoey, Naratip Sa-nguanpanich

**Affiliations:** ^1^Department of Research and Development, Faculty of Medicine Siriraj Hospital, Mahidol University, Bangkok, Thailand; ^2^Department of Physiology, Faculty of Medicine Siriraj Hospital, Mahidol University, Bangkok, Thailand; ^3^Department of Psychiatry, Faculty of Medicine Siriraj Hospital, Mahidol University, Bangkok, Thailand

**Keywords:** burnout syndrome, Maslach Burnout Inventory, reliability, validity, preclinical medical students

## Abstract

Burnout syndrome is characterized by emotional exhaustion, cynicism, and lack of professional efficacy. A considerable proportion of medical students experience burnout syndrome during their educational training. Therefore, this issue has become a major concern in the medical education community. The Maslach Burnout Inventory-Student Survey (MBI-SS) is the most widely used assessment of burnout syndrome among college students, including preclinical medical students. Therefore, our objective was to culturally modify and validate the MBI-SS in a Thai context for use with preclinical medical students. The MBI-SS comprises 16 items, including five items for emotional exhaustion, five items for cynicism, and six items for academic efficacy. Four hundred and twenty-six preclinical medical students participated in this study. We randomly divided the samples into two equivalent subsamples of 213 participants. The first subsample was used to calculate McDonald’s omega coefficients to assess internal consistency and to perform exploratory factor analysis. McDonald’s omega coefficients for exhaustion, cynicism, and academic efficacy were 0.877, 0.844, and 0.846, respectively. The scree plot from the unweighted least squares estimation and a direct oblimin rotation, supplemented with Horn’s parallel analysis and the Hull method, revealed three major factors of the Thai MBI-SS. Due to the violation of the multivariate normality assumption in the second subsample, we performed a confirmatory factor analysis with the unweighted least squares with a mean and variance adjusted estimation approach. The results of the confirmatory factor analysis showed favorable goodness-of-fit indices. Data from 187 out of 426 participants (43.9%), who completed a second questionnaire, were utilized to evaluate test–retest reliability. The correlation coefficients for test–retest reliability with a three-week period between tests were 0.724, 0.760, and 0.769 for the exhaustion, cynicism, and academic efficacy domains, respectively (all *p* < 0.05). This indicates that the Thai MBI-SS is a valid and reliable instrument to assess burnout syndrome in our Thai preclinical medical student population.

## Introduction

A study in 2018 estimated that 264 million people worldwide suffer from depression and depression is involved in 800,000 deaths annually (GBD 2017 Disease and Injury Incidence and Prevalence Collaborators, 2018). Healthcare workers, such as physicians, nurses and medical students, were reported to be at increased risk for depression and suicidal ideation (Mata et al., 2015; Rotenstein et al., 2016; Maharaj et al., 2018). Medical students are vulnerable to emotional distress through multiple stressors in various stages of their training.

Depression in medical students is complex and is intertwined with several factors ranging from anxious personal traits to relationship patterns (Silva et al., 2017). A study showed that burnout syndrome is closely related to depression in medical students, since these two entities share similar characteristics (Bianchi et al., 2015). Therefore, an improved understanding of burnout syndrome would improve both our understanding of depression and our ability to prevent depression in medical students. Burnout syndrome was defined as an occupational phenomenon caused by chronic stress related to work (Organization WH, 2018). Burnout syndrome is conceptualized in three dimensions, including emotional exhaustion, cynicism, and professional efficacy (Maslach and Jackson, 1981).

The Maslach Burnout Inventory-Student Survey (MBI-SS) is the most established and widely used tool to assess burnout syndrome in university students, including preclinical medical students (Dyrbye et al., 2010; Marôco and Campos, 2012; Campos et al., 2013). The MBI-SS comprises 16 items to evaluate three dimensions, including five questions for emotional exhaustion (EX), five questions for cynicism (CY), and six questions for academic efficacy (AE). The MBI-SS has been widely translated, culturally adapted, studied, and confirmed for validity and reliability in various countries that have different contextual and cultural settings (Schaufeli et al., 2002; Hu and Schaufeli, 2009; Campos et al., 2013; Yavuz and Dogan, 2014; Ilic et al., 2017; Portoghese et al., 2018). However, adaptation of the MBI-SS in a Thai university context is scarce. Consequently, the objective of this study was to culturally modify and validate MBI-SS for use in a Thai university context and especially for preclinical medical students. A valid and reliable Thai version of the MBI-SS would improve our ability to identify students with burnout syndrome, which would facilitate interventions designed to alleviate burnout and prevent depression.

## Methods

### Setting

This study was carried out at Faculty of Medicine Siriraj Hospital, Mahidol University, in Bangkok, Thailand. Siriraj Hospital is Thailand’s largest national tertiary referral center and serves the medical needs of approximately 3 million outpatients and 80,000 inpatients per year. Each year, approximately 320 high school graduates are enrolled in our 6-year undergraduate Doctor of Medicine degree program. Our curriculum consists of an introductory year in fundamental general science, 2 years of preclinical courses in basic medical science (preclinical years), 2 years of clinical rotations, and a final year of clinical clerkship (Wongtrakul and Dangprapai, 2020).

### Study design and study population

During February to March 2019, a total of 639 preclinical medical students (322 second-year students and 317 third-year students) were asked to participate voluntarily in this cross-sectional electronic questionnaire-based study. The questionnaires were completed online twice by each study participant – at baseline and then 3 weeks later (Ilic et al., 2017).

### Ethical considerations

The Human Research Protection Unit of the Institutional Review Board of our medical school (Ethics ID 170/2561; EC1) granted ethics approval for this cross-sectional study. Before participation, all study subjects gave their informed written consent.

### Instruments

Mind Garden, Inc. granted us permission to translate and culturally adapt the MBI-SS to develop the Thai version of the MBI-SS. Our translation and cultural modification of the MBI-SS were performed according to the guidelines of the World Health Organization and previously peer-reviewed articles (World Health Organization, 2015; Ilic et al., 2017). A linguistic expert and a qualified psychologist from our university independently conducted a translation from the English language to the Thai language. Subsequent discussions were held with a senior psychiatrist who has experience with burnout syndrome to settle disagreements in translation and finalize the translation. A pilot study in 20 preclinical students to evaluate the translated MBI-SS indicated that it was not difficult to understand any aspect of the questionnaire. A bilingual American linguistic professor from a different university performed the backward translation from Thai to English. A comparison between the original English-language version of the MBI-SS and the back-translated English version of the MBI-SS revealed no notable dissimilarities.

### Statistical analysis

Statistical Package for the Social Sciences software (SPSS, Inc., version 25, Chicago, IL, United States) was used for all statistical analysis for descriptive data.

To determine the validity of MBI-SS, we performed a cross-validation test to compute exploratory factor analysis (EFA), followed by confirmation factor analysis (CFA). To achieve cross-validation test, the total sample was randomly and equally divided into two distinct and equivalent subsamples using computerized randomization (Lorenzo-Seva, 2022). The first subsample was used for EFA, and the second subsample was used for CFA.

Before performing EFA in the first subsample, we determined whether our data were factorable (Williams et al., 2010; Lloret-Segura et al., 2014; Watkins, 2018). To test factorability, we conduct Kaiser-Mayer-Olkin (KMO) sampling adequacy measurement and Bartlett’s test of sphericity using the first subsample. The KMO value ranged from 0.00 to 1.00. A KMO value of equal to or greater than 0.70 was desired (Lloret et al., 2017). A KMO value of less than 0.50 was generally considered unacceptable, implying that the correlation matrix was not factorable (Kaiser, 1974). Bartlett’s test of sphericity was utilized to ensure that variables and factors did not overlap and should yield a significant level of p 0.05 to permit factorial analysis. The mean score plus/minus standard deviation (SD) for each item in the Thai MBI-SS of the first subsample was calculated. EFA was conducted on the first subsample, using the polychoric correlation matrix and the unweighted least squares (ULS) estimation method followed by a direct oblimin rotation using the FACTOR software version 12.02.01 (Lorenzo-Seva and Ferrando, 2006; González Casas et al., 2022). We calculated the McDonald’s omega coefficient for each factor to assess internal consistency. In addition, we calculated the McDonald’s omega coefficient for each domain if each item was removed. During EFA, the corrected item-total correlations of each item with its factor were calculated. If the corrected item-total correlations of each item with its factor were more than 0.50, it would be considered to have very good discrimination (Wickramasinghe et al., 2018). To identify the number of factors, we utilized the rule of Kaiser’s eigenvalues-greater-than-one and the scree plot, which were generated from ULS and a direct oblimin rotation. Furthermore, we supplemented the scree plot with Horn’s parallel analysis (Horn, 1965) and the Hull method (Lorenzo-Seva et al., 2011), which were the best methods to determine the number of factors (Lloret-Segura et al., 2014; Watkins, 2018). To assess the model fit indices in EFA, we calculated the chi-square (χ^2^/df), comparative fit index (CFI), Tucker-Lewis Index (TLI), and root mean square error of approximation (RMSEA) with 95% confidence interval (CI) and standardized root mean squared residual (SRMR) (Lloret-Segura et al., 2014; Lloret et al., 2017).

Before performing the CFA in the second subsample, we determined whether the Maximum Likelihood (ML) parameter estimation method should be used for our analysis by testing the multivariate normality of the data. We used Mardia’s test to obtain the multivariate coefficient of skewness and kurtosis (Mardia, 1970). If the assumption of multivariate normality was violated, we used the unweighted least squares (ULS) with mean and variance adjusted (ULSMV) estimation approach using MPLUS v. 7 software (Muthén and Muthén, 1998). To assess model fit indices in CFA, we calculated the chi-square, CFI, TLI, RMSEA and SRMR (Yu, 2002). For both EFA and CFA, if the RMSEA value is below 0.10, the CFI and TLI values are above 0.90, and the SRMR value is between 0.05 and 0.08, the model is considered to have a satisfactory fit (Campos et al., 2013; Cangur and Ercan, 2015). A value of *p* less than 0.05 was considered statistically significant for all tests.

To determine the reliability of the MBI-SS, we evaluated test–retest reliability in all participants who completed the questionnaire on two occasions 3 weeks apart (Ilic et al., 2017).

## Results

Four hundred and twenty-seven out of 639 preclinical medical students participated in this study (response rate: 66.8%); however, a medical student was excluded from an analysis due to his random response by giving the same answer (zero or never) on every item. Therefore, 426 participants were included in a final analysis, and 187 of 426 participants (43.9%) completed a second questionnaire response 3 weeks after their baseline questionnaire response. The sociodemographic characteristics of the study participants, including class year, sex, age, and hometown region, are summarized in Table 1. A total of 426 participants were randomly and equally divided into the first subsample of 213 participants for EFA and the second subsample of 213 participants for CFA.

**Table 1:** Summary of the sociodemographic data of the student participants.

Variable	Subsample 1 for EFA		Subsample 2 for CFA	
	*n*	%	*n*	%
Class year
- Year 2	113	53.1	113	53.1
- Year 3	100	46.9	100	46.9
Gender				
- Male	122	57.3	104	48.8
- Female	91	42.7	109	51.2
Age distribution (years)
- 19	44	20.7	33	15.5
- 20	95	44.6	98	46.0
- 21	70	32.9	72	33.8
- 22	4	1.9	7	3.3
Hometown region
- Bangkok	110	51.6	127	59.6
- Central	30	14.1	24	11.3
- North Eastern	5	2.3	9	4.2
- Northern	11	5.2	12	5.6
- Southern	8	3.8	7	3.3
- Eastern	32	15	19	8.9
- Western	17	8	15	7

### Exploratory factor analysis

**Figure 1:**
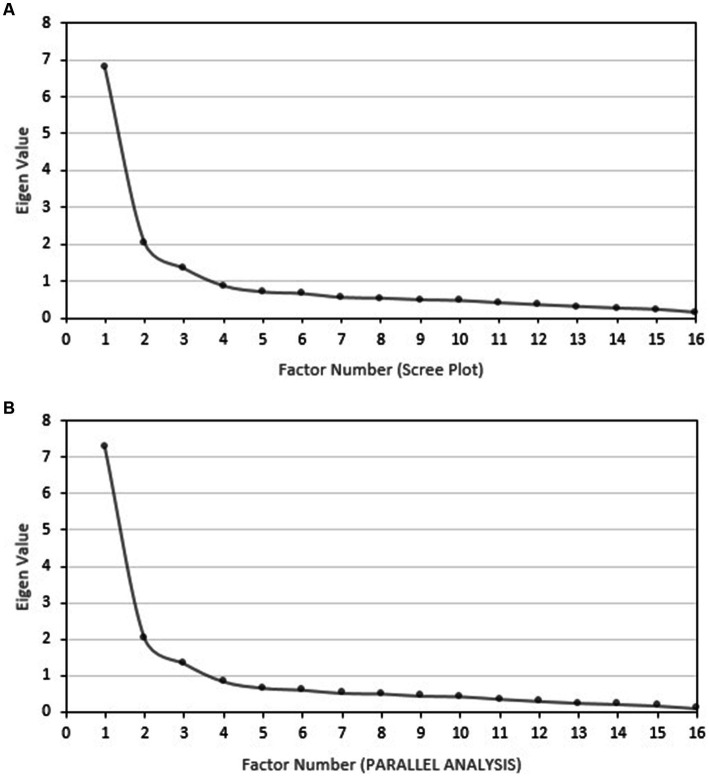
Factor identification methods used in EFA **(A)** scree plot and **(B)** parallel analysis.

The KMO value in the first subsample was 0.872, indicating factorability. Bartlett’s test of sphericity yielded a significant level of *p* < 0.001, χ^2^(120) = 2158.0 to permit EFA. Table 2 presents the mean score plus/minus standard deviation (SD), skewness, kurtosis, corrected item-total correlation of each item with its factor, and the factor loading of the analysis of the three-factor solution of the elements in the Thai MBI-SS of the first subsample during EFA. The McDonald’s omega coefficients in the EX, CY and AE domains were 0.877, 0.844 and 0.846, respectively. Table 2 also summarized the McDonald’s omega coefficients in each domain if each item was deleted. Interestingly, excluding each item resulted in lower McDonald’s omega coefficients of each domain than those from the 16-item version of that questionnaire. The corrected item-total correlations of each item with its factor were all positive, ranging from 0.56 to 0.75, supporting a very good discrimination of Thai MBI-SS. The polychoric correlation matrix was available in Supplementary Data S1. A scree plot, which was generated from ULS method with a direct oblimin rotation, confirms three major components of the Thai MBI-SS with an Eigenvalue greater than 1 (Figure 1A). Horn’s parallel analysis and the Hull method supported three major components of the Thai MBI-SS with an Eigenvalue greater than 1 (Figure 1B and Supplementary Data S2). The EFA results [χ^2^/df = 1.673 *p* < 0.001, CFI = 0.989, TLI = 0.983, RMSEA = 0.056 (95% CI, 0.055–0.065), and SRMR = 0.042] showed a good fit of the model.

**Table 2:** Descriptive data for each item of the Thai MBI-SS with the factor loading of the analysis of the three-factor solution of the elements in the first subsample of preclinical medical students at the Faculty of Medicine Siriraj Hospital, Mahidol University, Thailand.

Items	Factor loadings	McDonald’s omega	McDonald’s omega if the item is dropped	Corrected item-total correlation	Mean	SD	Skewness	Kurtosis
EX		0.877						
MBI1	0.820		0.839	0.75	3.87	1.45	−0.35	−0.72
MBI2	0.816		0.845	0.73	3.69	1.53	−0.65	−0.22
MBI3	0.793		0.846	0.73	3.76	1.73	−0.50	−0.60
MBI4	0.581		0.875	0.60	3.29	1.74	−0.17	−0.84
MBI6	0.732		0.849	0.71	3.09	1.66	−0.05	−0.73
CY		0.844						
MBI8	0.857		0.779	0.75	2.84	1.78	0.07	−0.97
MBI9	0.752		0.799	0.69	3.28	1.70	−0.17	−0.80
MBI13	0.607		0.835	0.56	3.93	1.62	−0.41	−0.67
MBI14	0.687		0.825	0.63	2.44	1.68	0.36	−0.80
MBI15	0.669		0.832	0.59	1.60	1.70	0.84	−0.33
AE		0.852						
MBI5	0.643		0.839	0.58	4.24	1.32	−0.63	−0.06
MBI7	0.650		0.835	0.60	3.23	1.69	−0.16	−0.84
MBI10	0.622		0.840	0.57	4.14	1.56	−0.72	−0.32
MBI11	0.721		0.827	0.64	4.51	1.31	−0.85	0.56
MBI12	0.778		0.815	0.70	3.82	1.36	−0.32	−0.26
MBI16	0.789		0.813	0.72	3.99	1.31	−0.59	0.27

MBI-SS, Maslach Burnout Inventory; EX, emotional exhaustion; CY, cynicism; AE, academic efficacy; SD, standard deviation.

### Confirmatory factor analysis

**Figure 2:**
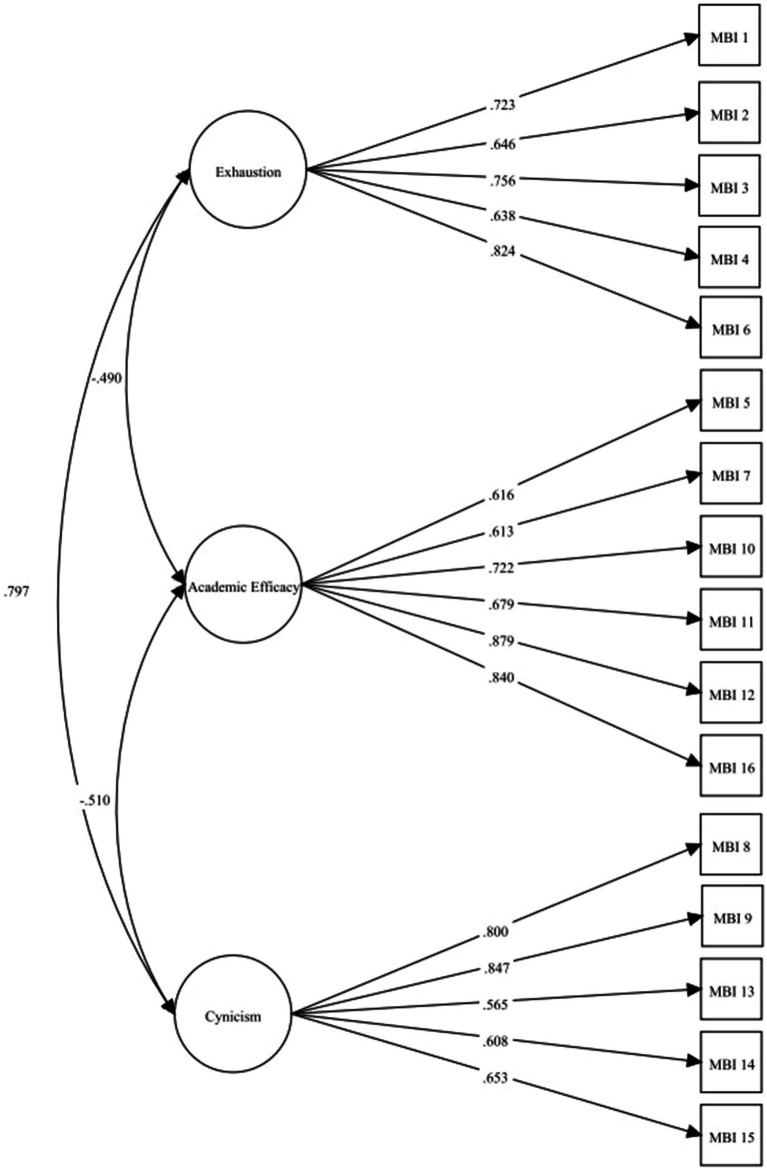
Confirmatory factor analysis of the Thai Maslach Burnout Inventory-Student Survey.

Mardia’s test reported the multivariate coefficient of skewness of 40.142 (*p* > 0.001) and kurtosis of 328.150 (*p* < 0.001) implying the violation of the assumption of multivariate normality. Therefore, we performed CFA with the ULSMV estimation approach. The path diagram shown in Figure 2 shows standardized coefficients of the relationship between factors and items. All factor loadings and the correlation between the factors in Figure 2 were statistically significant with a *p*-value <0.05. The CFA results [χ^2^/df = 1.813 *p* < 0.001, CFI = 0.964, TLI = 0.957, RMSEA = 0.062 (95% CI, 0.047–0.076), and SRMR = 0.055] demonstrated an acceptable fit of the model.

The correlation coefficients for test–retest reliability were 0.724, 0.760, and 0.769 for the EX, CY, and AE domains, respectively (all *p* < 0.05), indicating adequate reliability of the Thai MBI-SS.

## Discussion

This study in Thai preclinical medical students with extensive statistical analysis revealed good psychometric properties of the 16-item MBI-SS. The high McDonald’s omega coefficients of 0.877, 0.844, and 0.846 for the EX, CY, and AE domains, which indicate sufficient internal consistency of Thai MBI-SS, were similar to those of previously published studies of MBI-SS in non-English versions of MBI-SS, including Serbian, Sinhala (Sri Lanka), Chinese, Turkish, Iranian, Spanish, Portuguese, and Dutch (Schaufeli et al., 2002; Hu and Schaufeli, 2009; Rostami et al., 2013; Yavuz and Dogan, 2014; Ilic et al., 2017; Wickramasinghe et al., 2018).

The duration between the first and second take of the Thai MBI-SS questionnaire was 3 weeks, and the test–retest correlation coefficients were approximately 0.7 for the three domains, which are regarded as acceptable. Our results showed slightly higher reliability than those of the Serbian MBI-SS adaptation study (0.67–0.71) (Ilic et al., 2017). In contrast, a study of MBI-SS adaptation from Sri Lanka that had a two-week gap between the questionnaires showed markedly higher test–retest reliability (0.85–0.91) (Wickramasinghe et al., 2018).

The Thai MBI-SS, which consists of 16 items, showed high McDonald’s omega coefficients; however, other previous adaptations of the MBI-SS usually omitted one question for a total of 15 (five items in EX, four items in CY, and six in AE) (Marôco and Campos, 2012; Campos et al., 2013; Ilic et al., 2017; Wickramasinghe et al., 2018). Wickramasinghe et al. (2018) described their reasons for omitting item 13 (“I just want to get my work done and not be bothered”) as it lacked clarity and had poor psychometric properties. Although item 13 in our Thai MBI-SS showed the poorest psychometric property in exploratory factor analysis with a value of 0.607, it was not omitted due to the overall high McDonald’s omega coefficient observed in the CY subscale and was clearly interpreted as having a negative meaning in the Thai version. Additionally, if item 13 was deleted, McDonald’s omega coefficient in the CY subscale became 0.835, which was lower than 0.844 that was obtained from five items in the CY domain, which supports that no item should be removed.

Consistent with several studies, our Thai MBI-SS was better fitted to the 3-dimensional model (Hu and Schaufeli, 2009; Yavuz and Dogan, 2014; Faye-Dumanget et al., 2017; Ilic et al., 2017; Wickramasinghe et al., 2018). Our study confirmed not only the validity and reliability of the Thai MBI-SS, but also the importance of using reliable and standardized instruments, such as the MBI-SS, to conduct future research specific to burnout syndrome (Chirico et al., 2022).

Our validated Thai MBI-SS can and will be used to identify students with burnout syndrome and to evaluate the effects of several interventions with demonstrated preventive and alleviative effects on burnout syndrome, including health promotion programs that support physical activity, mindfulness, spirituality, and self-compassion (Naczenski et al., 2017; Li et al., 2019; Suleiman-Martos et al., 2020; Chirico et al., 2022). Interventions focusing on mindfulness and spirituality (Chirico, 2021) showed benefit even in the COVID-19 pandemic, which increased the burden on mental health in many different populations, including medical students (Zis et al., 2021).

### Strengths and limitations

Our study has several strengths. First, we enrolled the large number of preclinical medical students from Thailand’s largest medical school to participate in this study. Second, we performed a comprehensive statistical analysis and constructed models to determine the internal validity and reliability of the Thai MBI-SS.

Regarding the limitations of this study, we had a low retest rate of 43.9% and no external validation was conducted. We did not perform external validation of Thai MBI-SS in this study because MBI-SS is considered the gold standard for evaluating burnout syndrome.

## Conclusion

The Thai MBI-SS was shown to be a valid and reliable assessment of burnout syndrome in our Thai preclinical medical student population. An improved understanding of burnout syndrome in this vulnerable student population will accelerate intervention and improve educational and quality of life outcomes.

## Data availability statement

The raw data supporting the conclusions of this article will be made available by the authors, without undue reservation.

## Ethics statement

The studies involving human participants were reviewed and approved by The Human Research Protection Unit of the Siriraj Institutional Review Board, Faculty of Medicine Siriraj Hospital, Mahidol University, (1) Chairat Shayakul, M.D. Chairman of Ethics Committee, (2) Kitirat Techatraisak, M.D., Ph.D. Vice-Chairman of Ethics Committee, (3) Patcharin Tikhinanon, Administrator of Ethics Committee, E-mail: siethics@mahidol.ac.th. The patients/participants provided their written informed consent to participate in this study.

## Author contributions

WW: study design, data collection, and manuscript writing. YD: study design, data collection, and manuscript writing. NS: study design and manuscript writing. NS-n: statistical analysis and manuscript writing. All authors contributed to the article and approved the submitted version.

## Funding

This study was supported by the Faculty of Medicine Siriraj Hospital, Mahidol University (grant no. R016161008).

## Conflict of interest

The authors declare that the research was conducted in the absence of any commercial or financial relationships that could be construed as a potential conflict of interest.

## Publisher’s note

All claims expressed in this article are solely those of the authors and do not necessarily represent those of their affiliated organizations, or those of the publisher, the editors and the reviewers. Any product that may be evaluated in this article, or claim that may be made by its manufacturer, is not guaranteed or endorsed by the publisher.

## References

[ref1] Bianchi R. Schonfeld I. S. Laurent E. (2015). Burnout–depression overlap: a review. Clin. Psychol. Rev. 36, 28–41. doi: 10.1016/j.cpr.2015.01.004 25638755

[ref2] Campos J. A. D. B. Carlotto M. S. Marôco J. (2013). Copenhagen burnout inventory-student version: adaptation and transcultural validation for Portugal and Brazil. Psicol. Reflex. Crítica. 26, 87–97.

[ref3] Campos J. A. D. B. Jordani P. C. Zucoloto M. L. Bonafé F. S. S. Maroco J. (2013). Burnout in dental students: effectiveness of different methods. Rev. Odontol. UNESP 42, 324–329. doi: 10.1590/S1807-25772013000500002

[ref4] Cangur S. Ercan I. (2015). Comparison of model fit indices used in structural equation modeling under multivariate normality. J. Mod. Appl. Stat. Methods 14, 152–167. doi: 10.22237/jmasm/1430453580

[ref5] Chirico F. (2021). Spirituality to cope with COVID-19 pandemic, climate change and future global challenges. J. Health Soc. Sci. 6, 151–158. doi: 10.19204/2021/sprt2

[ref6] Chirico F. Nucera G. Leiter M. (2022). Measuring burnout syndrome requires reliable and standardized measures. Hong Kong J. Emerg. Med. 29, 325–326. doi: 10.1177/10249079221096920

[ref7] Dyrbye L. N. Massie F. S. Eacker A. Harper W. Power D. Durning S. J.. (2010). Relationship between burnout and professional conduct and attitudes among US medical students. JAMA 304, 1173–1180. doi: 10.1001/jama.2010.1318, PMID: 20841530

[ref8] Faye-Dumanget C. Carré J. Le Borgne M. Boudoukha P. A. H. (2017). French validation of the Maslach burnout inventory-student survey (MBI-SS). J. Eval. Clin. Pract. 23, 1247–1251. doi: 10.1111/jep.12771, PMID: 28653800

[ref9] GBD 2017 Disease and Injury Incidence and Prevalence Collaborators (2018). Global, regional, and national incidence, prevalence, and years lived with disability for 354 diseases and injuries for 195 countries and territories, 1990-2017: a systematic analysis for the global burden of disease study 2017. Lancet 392, 1789–1858. doi: 10.1016/S0140-6736(18)32279-7, PMID: 30496104 PMC6227754

[ref10] González Casas D. Barbé A. I. D. Mercado García E. Calleja Jimenez J. P. Gálvez-Nieto J. L. (2022). Psychometric examination of the freshman stress questionnaire using a sample of social work students in Spain during the COVID-19 pandemic. Br. J. Soc. Work 52, 4703–4720. doi: 10.1093/bjsw/bcac074

[ref11] Horn J. L. (1965). A rationale and test for the number of factors in factor analysis. Psychometrika 30, 179–185. doi: 10.1007/BF02289447 14306381

[ref12] Hu Q. Schaufeli W. B. (2009). The factorial validity of the Maslach burnout inventory–student survey in China. Psychol. Rep. 105, 394–408. doi: 10.2466/PR0.105.2.394-408, PMID: 19928601

[ref13] Ilic M. Todorovic Z. Jovanovic M. Ilic I. (2017). Burnout syndrome among medical students at one University in Serbia: validity and reliability of the Maslach burnout inventory—student survey. Behav. Med. 43, 323–328. doi: 10.1080/08964289.2016.1170662, PMID: 27127903

[ref14] Kaiser H. F. (1974). An index of factorial simplicity. Psychometrika 39, 31–36. doi: 10.1007/BF02291575

[ref15] Li C. Zhu Y. Zhang M. Gustafsson H. Chen T. (2019). Mindfulness and athlete burnout: a systematic review and meta-analysis. Int. J. Environ. Res. Public Health 16. doi: 10.3390/ijerph16030449 PMC6388258 30717450

[ref16] Lloret S. Ferreres A. Hernández A. Tomás I. (2017). The exploratory factor analysis of items: guided analysis based on empirical data and software. Anal. Psicol. 33, 417–432. doi: 10.6018/analesps.33.2.270211

[ref17] Lloret-Segura S. Ferreres-Traver A. Hernández-Baeza A. Tomás-Marco I. (2014). El análisis factorial exploratorio de los ítems: Una guía práctica, revisada y actualizada. [Exploratory Item Factor Analysis: A practical guide revised and updated.]. Anal. Psicol. 30, 1151–1169.

[ref18] Lorenzo-Seva U. (2022). SOLOMON: a method for splitting a sample into equivalent subsamples in factor analysis. Behav. Res. Methods 54, 2665–2677. doi: 10.3758/s13428-021-01750-y, PMID: 34918226 PMC9729132

[ref19] Lorenzo-Seva U. Ferrando P. J. (2006). FACTOR: a computer program to fit the exploratory factor analysis model. Behav. Res. Methods 38, 88–91. doi: 10.3758/BF03192753 16817517

[ref20] Lorenzo-Seva U. Timmerman M. E. Kiers H. A. (2011). The Hull method for selecting the number of common factors. Multivariate Behav. Res. 46, 340–364. doi: 10.1080/00273171.2011.564527, PMID: 26741331

[ref21] Maharaj S. Lees T. Lal S. (2018). Prevalence and risk factors of depression, anxiety, and stress in a cohort of Australian nurses. Int. J. Environ. Res. Public Health 16:61. doi: 10.3390/ijerph16010061 30591627 PMC6339147

[ref22] Mardia K. V. (1970). Measures of multivariate skewness and kurtosis with applications. Biometrika 57, 519–530. doi: 10.1093/biomet/57.3.519

[ref23] Marôco J. Campos J. A. D. B. (2012). Defining the student burnout construct: a structural analysis from three burnout inventories. Psychol. Rep. 111, 814–830. doi: 10.2466/14.10.20.PR0.111.6.814-830, PMID: 23402050

[ref24] Maslach C. Jackson S. E. (1981). The measurement of experienced burnout. J. Organ. Behav. 2, 99–113. doi: 10.1002/job.4030020205

[ref25] Mata D. A. Ramos M. A. Bansal N. Khan R. Guille C. Di Angelantonio E.. (2015). Prevalence of depression and depressive symptoms among resident physicians: a systematic review and Meta-analysis. JAMA 314, 2373–2383. doi: 10.1001/jama.2015.15845, PMID: 26647259 PMC4866499

[ref26] Muthén L Muthén B. Mplus user’s guide, 7th Edn Los Angeles. CA: Muthén & Muthén[Google Scholar] (1998)

[ref27] Naczenski L. M. Vries J. D. Hooff M. Kompier M. A. J. (2017). Systematic review of the association between physical activity and burnout. J. Occup. Health 59, 477–494. doi: 10.1539/joh.17-0050-RA, PMID: 28993574 PMC5721270

[ref28] Organization WH (2018). International classification of diseases. 11th Edn (ICD-11) Edn, 1–6.

[ref29] Portoghese I. Leiter M. P. Maslach C. Galletta M. Porru F. D’Aloja E.. (2018). Measuring burnout among university students: factorial validity, invariance, and latent profiles of the Italian version of the Maslach burnout inventory student survey (MBI-SS). Front. Psychol. 9:2105. doi: 10.3389/fpsyg.2018.02105, PMID: 30483171 PMC6240654

[ref30] Rostami Z. Abedi M. R. Schaufeli W. Ahmadi A. Sadeghi A. H. (2013). The psychometric characteristics of the Maslach burnout Invertory–student survey–among students of Isfahan university. Zahedan J. Res. Med. Sci. 15, 29–33.

[ref31] Rotenstein L. S. Ramos M. A. Torre M. Segal J. B. Peluso M. J. Guille C.. (2016). Prevalence of depression, depressive symptoms, and suicidal ideation among medical students: a systematic review and Meta-analysis. JAMA 316, 2214–2236. doi: 10.1001/jama.2016.17324, PMID: 27923088 PMC5613659

[ref32] Schaufeli W. B. Martinez I. M. Pinto A. M. Salanova M. Bakker A. B. (2002). Burnout and engagement in university students: a cross-national study. J. Cross-Cult. Psychol. 33, 464–481. doi: 10.1177/0022022102033005003

[ref33] Silva V. Costa P. Pereira I. Faria R. Salgueira A. P. Costa M. J.. (2017). Depression in medical students: insights from a longitudinal study. BMC Med. Educ. 17. doi: 10.1186/s12909-017-1006-0 PMC5633876 29017594

[ref34] Suleiman-Martos N. Gomez-Urquiza J. L. Aguayo-Estremera R. Cañadas-De La Fuente G. A. De La Fuente-Solana E. I. Albendín-García L. (2020). The effect of mindfulness training on burnout syndrome in nursing: a systematic review and meta-analysis. J. Adv. Nurs. 76, 1124–1140. doi: 10.1111/jan.14318, PMID: 32026484

[ref35] Watkins M. W. (2018). Exploratory factor analysis: a guide to best practice. J. Black Psychol. 44, 219–246. doi: 10.1177/0095798418771807

[ref36] Wickramasinghe N. D. Dissanayake D. S. Abeywardena G. S. (2018). Validity and reliability of the Maslach burnout inventory-student survey in Sri Lanka. BMC Psychol. 6:52. doi: 10.1186/s40359-018-0267-7, PMID: 30419960 PMC6233560

[ref37] Williams B. Onsman A. Brown T. (2010). Exploratory factor analysis: a five-step guide for novices. Austral. J. Paramed. 8, 1–13. doi: 10.33151/ajp.8.3.93

[ref38] Wongtrakul W. Dangprapai Y. (2020). Effects of live lecture attendance on the academic achievement of preclinical medical students. Med Sci Educ 30, 1523–1530. doi: 10.1007/s40670-020-01068-y, PMID: 34457820 PMC8368342

[ref39] World Health Organization. Process of translation and adaptation of instruments. (2015). Available at: http://www.who.int/substance_abuse/research_tools/translation/en/

[ref40] Yavuz G. Dogan N. (2014). Maslach burnout inventory-student survey (MBI-SS): a validity study. Procedia. Soc. Behav. Sci. 116, 2453–2457. doi: 10.1016/j.sbspro.2014.01.590

[ref41] Yu C.-Y. Evaluating cutoff criteria of model fit indices for latent variable models with binary and continuous outcomes: University of California, Los Angeles (2002)

[ref42] Zis P. Artemiadis A. Bargiotas P. Nteveros A. Hadjigeorgiou G. M. (2021). Medical studies during the COVID-19 pandemic: the impact of digital learning on medical Students' burnout and mental health. Int. J. Environ. Res. Public Health 18. doi: 10.3390/ijerph18010349, PMID: 33466459 PMC7796433

