# Supplementary material for: Reliability and validity study of the Thai adaptation of the Maslach Burnout Inventory-Student Survey among preclinical medical students at a medical school in Thailand
Source: Front Psychol. 2023 May 3;14:1054017. doi: 10.3389/fpsyg.2023.1054017 (PMC10190129; doi:10.3389/fpsyg.2023.1054017)
Supplement: Supplementary file 1 [file Table_1.DOCX]

STANDARIZED VARIANCE / COVARIANCE MATRIX (POLYCHORIC CORRELATION)

(Polychoric algorithm: Bayes modal estimation; Choi, Kim, Chen, & Dannels, 2011)

Variable 1 2 3 4 5 6 7 8 9 10 11 12 13 14 15 16

1 1.000

2 0.695 1.000

3 0.678 0.693 1.000

4 0.496 0.536 0.565 1.000

5 -0.399 -0.412 -0.343 -0.333 1.000

6 0.750 0.612 0.585 0.556 -0.447 1.000

7 -0.265 -0.284 -0.290 -0.295 0.477 -0.375 1.000

8 0.444 0.405 0.439 0.534 -0.305 0.662 -0.421 1.000

9 0.454 0.462 0.442 0.414 -0.272 0.625 -0.334 0.849 1.000

10 -0.247 -0.254 -0.217 -0.303 0.393 -0.295 0.440 -0.429 -0.418 1.000

11 -0.188 -0.181 -0.120 -0.269 0.427 -0.332 0.458 -0.396 -0.305 0.497 1.000

12 -0.275 -0.299 -0.282 -0.361 0.512 -0.382 0.539 -0.388 -0.426 0.538 0.695 1.000

13 0.500 0.436 0.382 0.429 -0.187 0.547 -0.224 0.523 0.536 -0.195 -0.262 -0.298 1.000

14 0.441 0.369 0.338 0.459 -0.299 0.540 -0.360 0.503 0.463 -0.292 -0.328 -0.372 0.505 1.000

15 0.264 0.217 0.249 0.377 -0.246 0.482 -0.378 0.579 0.458 -0.239 -0.386 -0.309 0.386 0.685 1.000

16 -0.304 -0.414 -0.309 -0.325 0.596 -0.397 0.584 -0.429 -0.412 0.545 0.588 0.591 -0.277 -0.418 -0.403 1.000
