# Supplementary material for: Reliability and validity study of the Thai adaptation of the Maslach Burnout Inventory-Student Survey among preclinical medical students at a medical school in Thailand
Source: Front Psychol. 2023 May 3;14:1054017. doi: 10.3389/fpsyg.2023.1054017 (PMC10190129; doi:10.3389/fpsyg.2023.1054017)
Supplement: Supplementary file 2 [file Table_2.DOCX]

**HULL METHOD FOR SELECTING THE NUMBER OF COMMON FACTORS (HULL)**

Lorenzo-Seva, Timmerman, Kiers (2011)

Number of Goodness-of-fit Degrees of Scree test

factors values freedom values

0 0.128 120 0.000

1 0.297 104 1.524

2 0.401 89 1.485

3 0.466 75 2.901*

4 0.487 62 0.000

* Advised number of common factors: 3

**PARALLEL ANALYSIS (PA) (Horn, 1965)**

Variable Real-data Mean of random 95 percentile of random

eigenvalues eigenvalues eigenvalues

1 7.26821 1.50210 1.59195

2 2.04096 1.38927 1.45823

3 1.33705* 1.30807 1.37033

4 0.83848 1.23627 1.29234

5 0.65680 1.17572 1.22131

6 0.60609 1.11417 1.15783

7 0.51491 1.05840 1.10063

8 0.50286 1.00646 1.04674

9 0.43852 0.95281 0.99369

10 0.42553 0.90377 0.94319

11 0.35417 0.85415 0.89634

12 0.29617 0.80446 0.84656

13 0.24327 0.75652 0.79361

14 0.21211 0.70525 0.75039

15 0.16956 0.64998 0.69607

16 0.09530 0.58261 0.63893

* Advised number of dimensions when mean is considered: 3
